# PD-1 and CTLA-4 up regulation on donor T cells is insufficient to prevent GvHD in allo-HSCT recipients

**DOI:** 10.1371/journal.pone.0184254

**Published:** 2017-09-27

**Authors:** Mohammad S. Hossain, Ghada M. Kunter, Vicky F. El-Najjar, David L. Jaye, Zaid Al-Kadhimi, Owonikoko K. Taofeek, Jian-Ming Li, Edmund K. Waller

**Affiliations:** 1 Department of Hematology and Medical Oncology, Division of Stem Cell and Bone Marrow Transplantation, Winship Cancer Institute, Emory University School of Medicine, Atlanta, Georgia, United States of America; 2 Holzer Cancer Center, Gallipolis, Ohio, United States of America; 3 Department of Pathology, Emory University School of Medicine, Atlanta, Georgia, United States of America; Goethe University Medical School, GERMANY

## Abstract

The expression of checkpoint blockade molecules PD-1, PD-L1, CTLA-4, and foxp3+CD25+CD4+ T cells (Tregs) regulate donor T cell activation and graft-vs-host disease (GvHD) in allogeneic hematopoietic stem cell transplant (allo-HSCT). Detailed kinetics of PD-1-, CTLA-4-, and PD-L1 expression on donor and host cells in GvHD target organs have not been well studied. Using an established GvHD model of allo-HSCT (B6 → CB6F1), we noted transient increases of PD-1- and CTLA-4-expressing donor CD4+ and CD8+ T cells on day 10 post transplant in spleens of allo-HSCT recipients compared with syngeneic HSCT (syn-HSCT) recipients. In contrast, expression of PD-1- and CTLA-4 on donor T cells was persistently increased in bone marrow (BM) of allo-HSCT recipients compared with syn-HSCT recipients. Similar differential patterns of donor T cell immune response were observed in a minor histocompatibility (miHA) mismatched transplant model of GvHD. Despite higher PD-1 and CTLA-4 expression in BM, numbers of foxp3+ T cells and Tregs were much lower in allo-HSCT recipients compared with syn-HSCT recipients. PD-L1-expressing host cells were markedly decreased concomitant with elimination of residual host hematopoietic elements in spleens of allo-HSCT recipients. Allo-HSCT recipients lacking PD-L1 rapidly developed increased serum inflammatory cytokines and lethal acute GvHD compared with wild-type (WT) B6 allo-HSCT recipients. These data suggest that increased expression of checkpoint blockade molecules PD-1 and CTLA-4 on donor T cells is not sufficient to prevent GvHD, and that cooperation between checkpoint blockade signaling by host cells and donor Tregs is necessary to limit GvHD in allo-HSCT recipients.

## Introduction

Donor T-lymphocyte infusion can be an effective form of adoptive immunotherapy in the context of allo-HSCT, but life threatening complications related to GvHD limit its clinical application. Removal of donor T cells from the graft reduces GvHD but increases the incidences of graft failure, opportunistic infection, and tumor relapse [1–3]. Immunosuppressive drugs are commonly used to control GvHD, but have incomplete efficacy, and are frequently associated with drug-related toxicities and mortality [4]. Therefore, modulating donor T cell activity to enhance immune response against opportunistic infection and against tumor relapse in allo-HSCT recipients without increasing GvHD remains a long-standing goal. Programmed death-1 (PD-1) and cytotoxic T-lymphocyte antigen-4 (CTLA-4) expression negatively regulate T cell activity and lack of their expression leads to autoimmune diseases [5–9]. Therefore, *in vivo* immune modulation of donor T cells through PD-1 and CTLA-4 signaling pathways may play an important role in controlling GvHD in allo-HSCT recipients. Detailed kinetic studies of PD-1 and or CTLA-4 expression on donor CD4+ and CD8+ T cells and the kinetics of inducible PD-L1 expression on host cells and donor cells in GvHD target organs have not been done.

PD-1 has two known ligands, PD-L1 and PD-L2. PD-L1 binding to PD1 has a greater negative regulatory effect on T cells than does its interaction with PD-L2 [10–14]. PD-L1 is widely expressed on hematopoietic and non-hematopoietic cells and its expression is upregulated in the presence of immune stimuli [15]. Functional signaling through PD-1 is critical to limit GvHD in allo-HSCT, since blockade of PD-1 or PD-L1 with monoclonal antibody (mAb) increases GvHD toxicity and PD-L1 expression in host tissues is required to reduce GvHD [16, 17]. *In vivo* PD-L1 blockade increases CD4+ T cell-mediated skin allograft rejection and prevents apoptosis of alloantigen-specific effector T cells [18]. In addition to PD-1 signaling, inhibitory signaling through CTLA-4 binding to CD80/CD86 limits GvHD. CTLA-4 blocks the CD28-B7 mediated co-stimulatory pathway and inhibits donor T cell allo-reactivity in allo-HSCT recipients [7]. Deficiency of CTLA-4 in Tregs causes spontaneous development of systemic lymphoproliferation, fatal T cell–mediated autoimmune disease, and hyperproduction of immunoglobulin E in mice [19]. Administration of antibodies against CTLA-4 immediately after allo-HSCT was shown to increase acute GvHD, while the blocking of CTLA-4 at late phases of allo-HSCT did not increase GvHD but resulted in lethal lympho-splenomegaly, autoimmune hepatitis and increased circulating anti-DNA auto-antibodies [9]. In addition to the role of CTLA-4 and PD-1 in regulating allo-reactivity and GvHD, foxp3-expressing inducible donor Tregs and/or the addition of *in vitro* expanded donor Tregs to the graft have shown promising therapeutic benefit in the prevention of GvHD [20, 21].

In this study, we have explored the kinetics of expression of PD-1 and CTLA-4 by donor CD4+ T and CD8+ T cells in the spleen and BM, two hematolymphoid organs that are a target for GvHD. We show herein that, despite the abundant expression of inducible PD-1 and CTLA-4 by donor CD4+ and CD8+ T cells, and inducible PD-L1 expression by both host and donor cells in lethally irradiated allo-HSCT recipients, expression of these molecules failed to prevent the development of severe GvHD in allo-HSCT recipients. Importantly, up-regulation of PD-1 and CTLA-4 on donor CD4+ and CD8+ T cells were differentially regulated in spleen versus the BM of allo-HSCT recipients. The numbers of PD-L1 expressing host spleen cells markedly decreased over time, concomitant with the induction of GvHD in allo-HSCT recipients. PD-L1 KO allo-HSCT recipients had lethal acute GvHD associated with increased serum inflammatory cytokines, increased numbers of activated donor CD4+ and CD8+ T cells expressing ICOS-1, CD69 and PD-1 and decreased numbers of Tregs in the spleen compared with WT B6 allo-HSCT recipients. Our data highlight the differential kinetics of PD-1 and CTLA-4 expression by donor T cells in hematolymphoid tissues of allo-HSCT recipients and their role as potential targets for therapeutic intervention.

## Materials and methods

### Mice

CB6F1 (C57BL/6 X BALB/c)(H-2^b/d^, CD45.2/Thy1.2), B6.PL(H-2^b^, CD45.2/Thy1.1) and PepBoy (H-2^b^, CD45.1/Thy1.2) mice on the C57BL/6 background, and B10.BR (H-2^K^, CD45.2/Thy1.2) and BA.B10 (H-2^K^, CD45.2/Thy1.1) were purchased from Jackson Laboratories (Bar Harbor, ME). PD-L1 KO and PD-L2 KO mice in the C57BL/6 background (obtained from Rafi Ahmed Lab) were bred at Emory. All animal experiments and procedures conformed to *the Guide for the Care and Use of Laboratory Animals*, and were approved by the Emory University Institutional Animal Care and Use Committee.

### Preparation of donor splenocytes and BM from donor mice

For B6 → CB6F1 allo- and syn-HSCT models, splenocytes were obtained from PepBoy B6 congenic mice and cultured overnight at 10^7^ cells/ml in complete media at 37°C in 5% CO_2_ [22] to deplete adherent cells, particularly monocytes. Live cells were counted under fluorescence microscopy using ethidium bromide mixed with acridine orange dye [23]. Following overnight culture the cells were washed once and transplanted via intravenous injection. Bone marrow (BM) was flushed from femora and tibia of naïve CD45.2+Thy1.1+ B6 congenic donor mice and CD3+ T-cells were depleted (TCD) as described [24]. For the B10.BR → PD-L1 KO B6, PD-L2 KO B6 or WT B6 allo-HSCT models, splenocytes from naïve B10.BR mice were harvested, cultured overnight and T cells were further enriched for hematopoietic stem cells by depleting CD11b+CD11c+CD19+ cells using a Macs column. BM cells were harvested from naïve BA.B10 mice and hematopoietic stem cells were enriched by depleting CD3+ CD11b+ CD11c+ CD19+ cells using a MACS separation column as described previously [24].

### Irradiation and HSCT

On day –1, WT B6, CB6F1, PD-L1KO or PD-L2 KO mice were conditioned with a total of 11Gy irradiation divided into two doses (5.5 Gy each) 3 hours apart [25]. For the B6 → CB6F1 allo- and syn-HSCT models, 5 x 10^6^ TCD BM cells were transplanted along with 7.5 x 10^6^ donor splenocytes via tail vein injection into irradiated CB6F1 (allo-HSCT) or B6 (syn-HSCT) recipient mice. To minimize the contribution of immune interaction of PD-1/PD-L1 from donor cells in PD-L1 KO, PD-L2 KO or control WT B6 allo-HSCT recipients we depleted donor antigen-presenting cells and transplanted lower numbers of stem cell enriched BM and T cell enriched splenocytes. In B10.BR → PD-L1 KO, PD-L2 KO or WT B6 allo-HSCT model, 2 x 10^6^ stem cell-enriched BM cells were transplanted along with 2 x 10^6^ T cell-enriched donor splenocytes via tail vein injection into irradiated PD-L1 KO, PD-L2 KO or WT B6 mice. In the minor MHC mismatch C3H.SW→B6 transplant model [26], 11 Gy irradiated PepBoy (H-2^b^, CD45.1/Thy1.2) recipients received 5 x 10^6^ TCD BM cells from B6 GFP donor mice and 5 x 10^6^ splenocytes from C3H.SW (MiHA) donor mice. 11 Gy irradiated PepBoy (H-2^b^, CD45.1/Thy1.2) recipients received 5 x 10^6^ TCD BM cells from B6 GFP donor mice and 5 x 10^6^ splenocytes from congenic B6 (H-2^b^, CD45.2/Thy1.2) donor mice and were used as syngenic (no GvHD) control.

### Lymphocyte isolation from the spleen and BM of allo- and syn-HSCT recipients

Splenocytes were harvested from allo-and syn-HSCT recipients by gently crushing tissue between frosted glass slides (Fisher Scientific, Pittsburgh, PA, USA) [25]. BM cells were harvested from the tibia and femur of one hind leg as described before [27]. Single cell suspensions were prepared by passing the harvested cell suspension through a cell strainer (Becton Dickinson, Franklin Lakes, NJ, USA). Live lymphocytes were quantitated by fluorescence microscopy as previously described [24].

### Assessment of acute GvHD

Histological acute GvHD scores were determined on formalin-fixed liver and intestinal tissue sections independently reviewed by two experienced pathologists (D.L.J and V.F.E). Combined acute GvHD scores were calculated by measuring the weight loss (0–2), posture (0–2), activity (0–2), fur texture (0–2), skin integrity (0–2) and histological acute GvHD scores of liver (0–1), small intestine (0–4) and large intestine (0–4) as previously described [28]. Briefly, for intestinal tissue sections, Grade 1: presence of single apoptotic epithelial cells; Grade 2: presence of crypt abscesses; Grade 3: focal mucosal erosion; Grade 4: diffuse mucosal erosion. Grading of GvHD in the liver was assigned as either Grade 1 (bile duct epithelium apoptosis or presence of dense portal or multifocal lobular lymphoplasmacytic infiltrates) or Grade 0 (no evidence of GvHD). Microscopic images of tissue sections were taken at 200X using a Nikon Eclipse E400 microscope (Nikon, Melville, New York) using SPOT Flex 15.2 64 Mp Shifting Pixel Camera and SPOT software (Diagnostic Instruments Inc., Sterling Heights, MI).

### Flow cytometry

Flow cytometry was performed as previously described [24]. The origin of CD8+ and CD4+ T cells in allo-HSCT recipients was determined by staining with mAbs specific for donor spleen (CD45.1+), donor BM (CD45.1-H-2^d^-) or host T-cells (CD45.1-H-2^d^+) in combination with mAbs to CD8, CD4, PD-1, PD-L1, CTLA-4, ICOS-1, CD69, etc. Foxp3 transcription factor-expressing T cells were determined through intracellular staining using mouse Foxp3 kit purchased from eBioscience (San Diego, CA). Stained cells were acquired by FACS Canto (Becton Dickinson, San Jose, CA) and analyzed using FlowJo software. All antibodies were purchased from either BD Pharmingen (San Jose, CA) or eBiosciences.

### Serum cytokines measured by Luminex assay

Serum was harvested on day 8 after transplantation from PD-L1 KO and B6 allo-HSCT recipient mice. Mouse 26-plex kits were purchased from Affymetrix Inc (Santa Clara, CA) and the Luminex assay (Luminex Corp., Austin, Texas) was performed in a blinded fashion at the Immunology Core Laboratory at Stanford University (Stanford, CA) according to the manufacturer’s recommendations. All samples were assayed in a single batch, and each sample was measured in duplicate. Plates were read using a Luminex 200 instrument (Luminex Corp) as previously described [28].

### Statistical analyses

Mean values obtained from 4 to 6 mice per group were compared using two-sided Student’s *t*-test. Differences were considered significant when *p* values below the 0.05 levels were obtained.

## Results

### Increased expression PD-1 and CTLA-4 on donor CD4+ and CD8+ T cells is inadequate to reduce GvHD in allo-HSCT recipients

Previous work using *in vivo* imaging has shown that donor T cells migrate to almost all body compartments (GvHD target organs and other tissues) within 72 hours of transplantation and signaling through PD-1 and CTLA-4 play important roles in the regulation of allo-reactive donor T cells that cause GvHD [7] [29]. Using an established model of sub-acute GvHD (B6 → CB6F1) in which allo-HSCT recipients experienced 30% mortality and 20% weight loss by 100 days post transplant (S1 Fig), we first investigated the kinetics of PD-1, CTLA-4, PD-L1 and foxp3 expression on donor and host cells in hematolymphoid organs that are a target for GvHD. Allo-HSCT recipients were compared with syn-HSCT (B6 → B6) recipient mice transplanted with equivalent numbers of cells (no GvHD control; S1 Fig). Overall, hematopoiesis was suppressed in allo-HSCT recipients with GvHD. While the numbers of total splenocytes were similar on day 4 post transplant between allo- and syn-HSCT recipients (S2 Fig), the numbers of splenocytes in allo-HSCT recipients were significantly decreased (p<0.005) on days 10, 25 and 103 post transplant compared with syn-HSCT recipients (S2 Fig). The numbers of leukocytes harvested from BM in allo-HSCT recipients were higher on day 4, lower on days 10 and 25, and equivalent on day 103 post transplant compared with syn-HSCT recipients (S2 Fig). Allo-HSCT recipients also had fewer cells in the blood and thymus compared with syn-HSCT recipients (S2 Fig). The numbers of CD45.1+ donor cells were significantly expanded in the spleen and BM on day 10 post transplant in allo-HSCT recipients compared with syn-HSCT recipients but were decreased in the spleen (but not in BM) at subsequent time points concomitant with the development of sub-acute GvHD (S2 Fig). These data suggest that the immune responses of donor T cells in allo-HSCT recipients are organ specific and that allo-reactive donor T cells have greater suppressive effects on hematopoietic reconstitution in the spleen and thymus than the BM.

**Fig 1:**
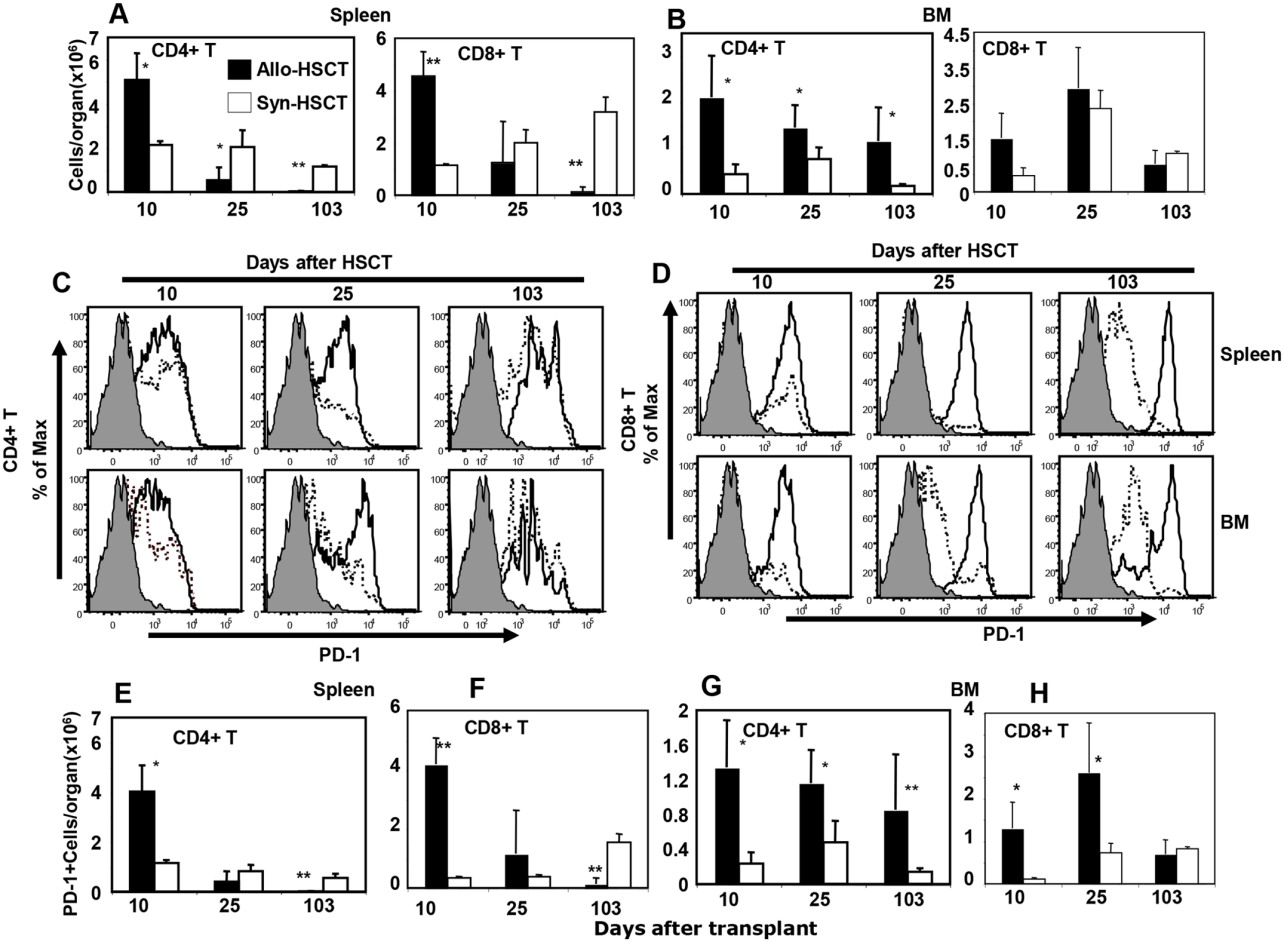
Differential expression of PD-1 by donor CD4+ and CD8+ T cells in spleen and BM of allo-HSCT recipients. A and B. Absolute numbers of donor spleen-derived (CD45.1+ gated) CD4+ and CD8+ T cells harvested from spleen and BM, respectively on day 10, 25 and 103 after transplant of B6 → CB6F1 allo-HSCT (filled bars) and B6 → B6 syn-HSCT (open bars) recipients. C and D represent the numbers of PD-1 expressing donor spleen-derived CD4+ and CD8+ gated T cells, respectively, harvested from the spleen and BM of allo-HSCT (solid lines) and syn-HSCT (dashed lines) recipients. Gray filled histograms are isotype controls. E and F represent the absolute numbers of donor spleen-derived PD-1 expressing CD4+ and CD8+ T cells per spleen, respectively after allo-HSCT (filled bars) and syn-HSCT (open bars) recipients. G and H represent the kinetics of absolute numbers of donor spleen-derived CD4+ and CD8+ T cells per BM, respectively, after allo-HSCT (filled bars) or syn-HSCT (open bars). The symbols “*” and “**”represent the *p* values <0.05 and <0.005, respectively, Students *t*-Test. The data are the representative of two independent experiments. Five mice were used per group in each time point.

To explore the basis for the differences in inflammation seen in the various hematolymphoid organs we studied the kinetics of PD-1 expression on donor T cells during sub-acute GvHD. Donor CD4+ and CD8+ T cells recovered from the spleen and BM of allo-HSCT recipients were phenotyped by flow cytometry and compared with T cells from syn-HSCT recipients. Numbers of CD45.1+ donor CD4+ and CD8+ donor T cells were significantly higher in the spleens of allo-HSCT recipients on day 10 after transplant but were lower at later time points compared with syn-HSCT recipients (Fig 1A). On the other hand, the numbers of CD45.1+ donor CD4+ T cells (but not CD8+ T cells) were higher at all time points in the BM of allo-HSCT recipients compared with syn-HSCT recipients (Fig 1B). These data suggested the hypothesis that local inflammation caused by GvHD in hematolymphoid organs is differentially regulated in the spleen versus the BM according to the immunological state of donor T cells at each site. To address this question, we measured the kinetics of PD-1 expression on CD4+ and CD8+ T cells in the spleen and BM of allo- and syn-HSCT recipients. Higher percentages of CD4+ T cells expressing PD-1 were detected in the spleen and BM on days 10 and 25 (but not on day 103) in allo-HSCT recipients compared with syn-HSCT recipients (Fig 1C). The percentages of PD-1 expressing CD8+ donor T cells in both spleen and BM were higher at all times post transplant in allo-HSCT recipients compared with syn-HSCT recipients (Fig 1D). Accordingly, the absolute numbers of PD-1 expressing donor CD4+ and CD8+ T cells were significantly increased on day 10 post transplant in spleen of allo-HSCT recipients but equivalent or lower at subsequent time points compared with syn-HSCT recipients (Fig 1E and 1F). In contrast, the numbers of PD-1 expressing donor CD4+ T cells were persistently higher at all time points in the BM of allo-HSCT recipients compared with syn-HSCT (Fig 1G) and numbers of PD-1 expressing donor CD8+ T cells were higher only on days 10 and 25 (but not on day 103) after transplant (Fig 1H). These data suggest that the kinetics of PD-1 expression on donor CD4+ and CD8+ T cells is differentially regulated in spleen versus BM.

**Fig 2:**
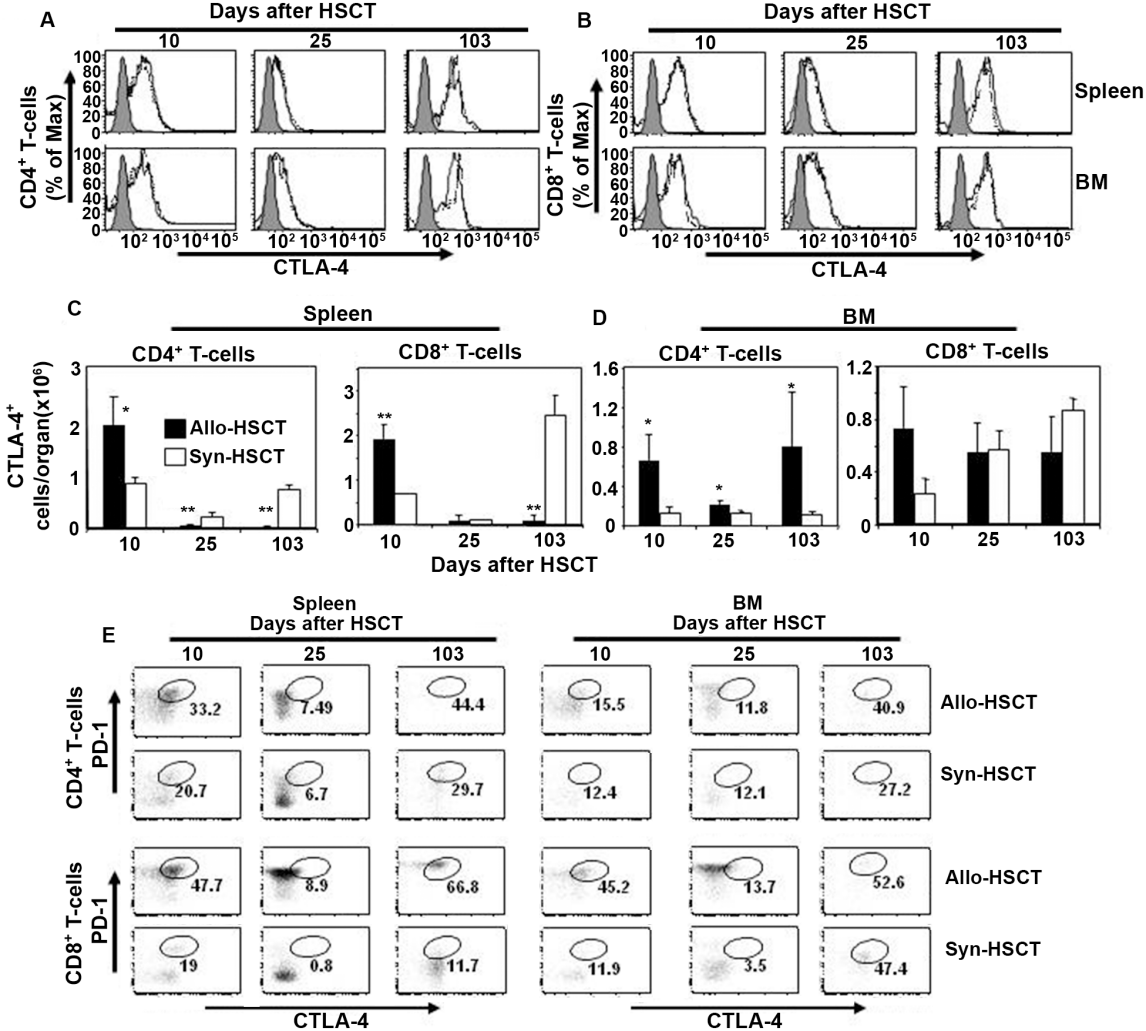
Differential expression of CTLA-4 by donor T cells in spleen and BM of allo-HSCT recipients. A and B represent the kinetics of CTLA-4 expression by donor spleen-derived (CD45.1+ gated) CD4+ and CD8+ T cells, respectively, harvested from spleen and BM on day 10, 25 and 103 after transplant of allo-HSCT (solid lines) and syn-HSCT (dashed lines) recipients. Gray filled histograms are the isotype controls. C. Numbers of total donor spleen-derived (CD45.1+ gated) CTLA-4+CD4+ and CTLA-4+CD8+ T cells per spleen in allo-HSCT (filled bars) and syn-HSCT (open bars) recipients. D. Donor spleen-derived (CD45.1+ gated) CTLA-4+CD4+ and CTLA-4+CD8+ T cells per BM (harvested from the tibia and femur of one hind leg) after transplant of allo-HSCT (filled bars) and syn-HSCT (open bars) recipients. E. Representative FACS plots of donor spleen-derived CTLA-4+PD-1+CD4+ T cells (upper two rows) and CTLA-4+PD-1+CD8+ T cells (lower two rows) harvested from spleen and BM after transplant of allo-HSCT and syn-HSCT recipients. 5 mice were used per group. The symbols “*” and “**” represent *p* values <0.05 and <0.005, respectively, Students *t*-Test. The data are the representative of two independent experiments.

We next determined the kinetics of CTLA-4 expression on donor CD4+ and CD8+ T cells in the spleen and BM of allo- and syn-HSCT recipients. Similar percentages of CTLA-4 expression were detected in donor CD4+ (Fig 2A) and CD8+ (Fig 2B) T cells in the spleen and BM of allo- and syn-HSCT recipients at all three time points post transplant. However, a transient increase in the absolute numbers of CTLA-4 expressing CD4+ and CD8+ T cells was seen on day 10 after transplant in the spleen of allo-HSCT compared with syn-HSCT recipients (Fig 2C). On the other hand, absolute numbers of CTLA-4 expressing CD4+ T cells (but not CD8+ T cells) in the BM of allo-HSCT recipients were significantly increased at all time points after transplant compared with syn-HSCT (Fig 2D). These data suggest that higher levels of CTLA4-expressing donor T cells may be responsible for limiting GvHD-associated inflammation in the BM (but not the spleen) of allo-HSCT recipients.

**Fig 3:**
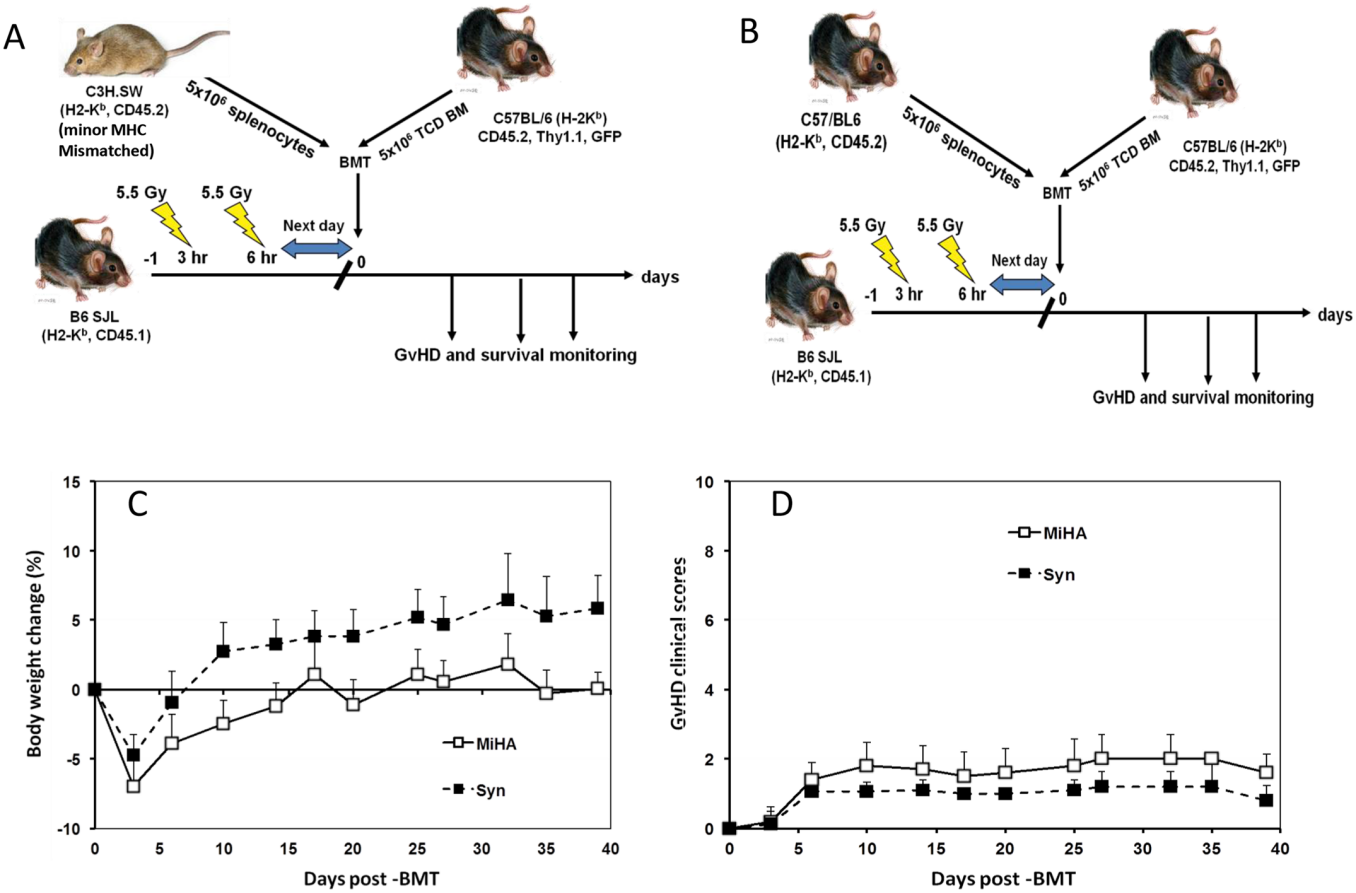
MiHA mismatched HSCT recipients showed minimal GvHD lethality without mortality. Recipient mice received 5 million BM cells from B6 GFP donor mice and 5 million splenocytes from either C3H.SW (miHA) donor or B6 (Syn) donor. A total of 15 were used each transplant group. A. miHA mismatched mouse models of transplantation. B. Syngenic mouse models of transplantation. C. Bodyweight loss of the recipient mice. D. Clinical score of acute GvHD in the transplanted mice.

**Fig 4:**
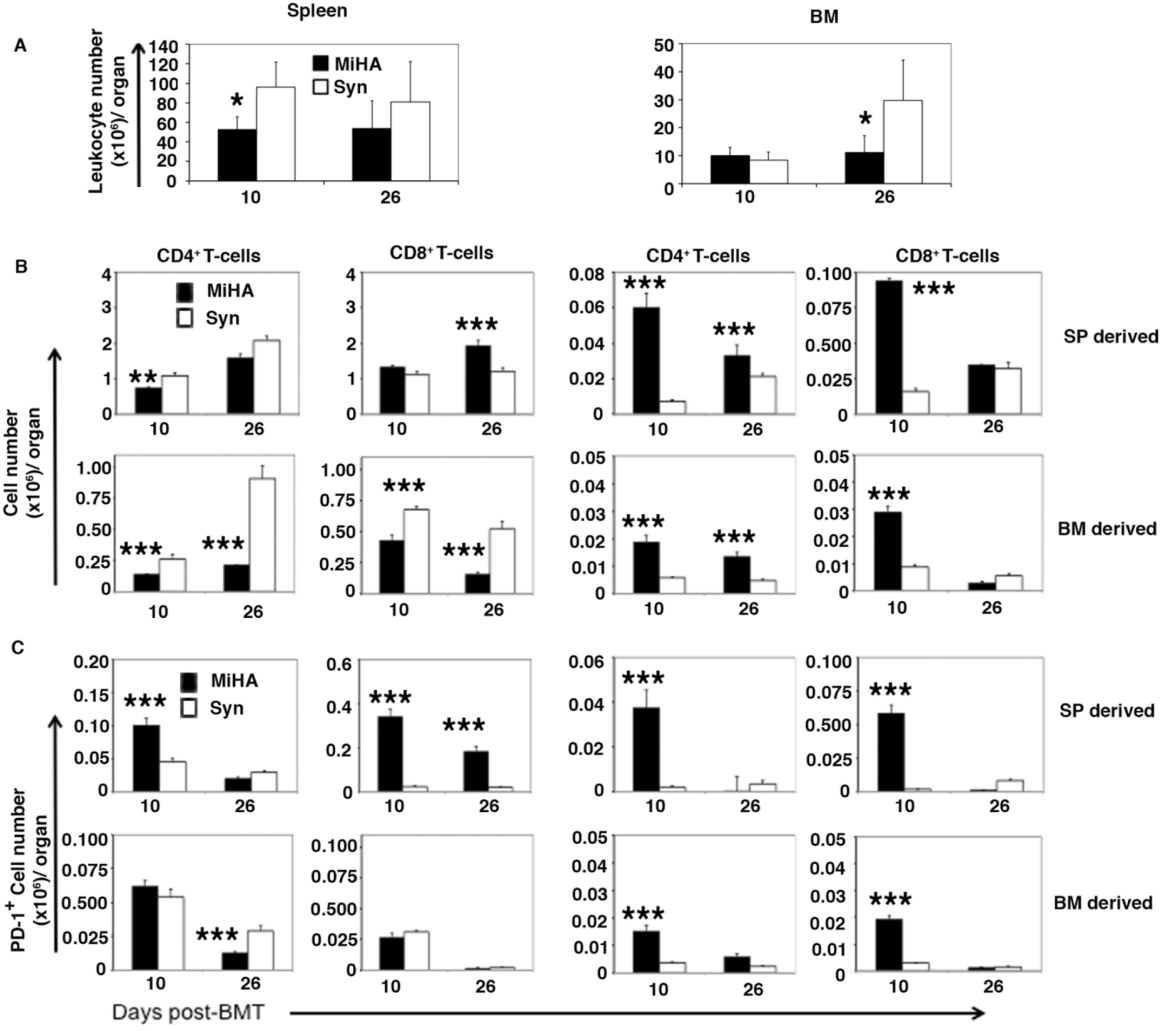
miHA allo-HSCT caused differential PD-1 expression by donor T cells in spleen and BM. Recipient mice received equal numbers (5 million) BM cells from B6 GFP donor mice and splenocytes from either C3H.SW (MiHA) donor or B6 (Syn) donor. N = 5 for each transplant group. The recipients were euthanized on 10 and 26 days post-HSCT. Surface markers were measured by flow cytometry and analyzed by FlowJo software. A. Overall leukocyte numbers in BM and spleen. B. Donor BM derived (CD45.2^+^GFP^+^) or donor splenocyte derived (CD45.2^+^GFP^-^) CD4^+^ and CD8^+^ T-cells harvested from the recipients’ bone marrow and spleens. C. PD-1 expressing donor BM derived (CD45.2^+^GFP^+^) or donor splenocyte derived (CD45.2^+^GFP^-^) CD4^+^ and CD8^+^ T-cells harvested from the recipients’ bone marrow and spleens. * p<0.05; ** P<0.005; *** p<0.001 signify significant difference between MiHA and syngeneic transplant groups (Students *t*-Test).

Next, we investigated whether similar increased expression of PD-1 and CTLA-4 by donor T cells also occurs in a minor histocompatibility (miHA)-mismatched mouse GVHD model (Fig 3A) which may more closely resemble clinical GvHD seen in human allo-HSCT patients [26]. To address this we used the CD8+ T cell dependent C3H.SW→B6 miHA GvHD model which has minimal early lethality allowing collection of data from mice of multiple time points on after BMT [26, 30]. As anticipated, all recipient mice receiving 5 million donor splenocytes from C3H.SW donor mice survived with only slight delays in bodyweight gain and without significantly increased GvHD clinical scores compared with the syngeneic control recipients (Fig 3B and 3D). Total numbers of leukocytes were significantly lower (p<0.05) on day 10 in spleen and on day 26 in BM in miHA recipients compared with the syngenic control recipients (Fig 4A). Consistent with CD8+ T-cell mediated GVHD in this model, numbers of CD8+ T cells were higher in spleen on day 26 after allo-HSCT (Fig 3B) while numbers of donor spleen-derived CD4+ T cells in the spleen were significantly lower on day 10 compared with syn-HSCT recipients. In contrast, examination of BM showed higher numbers of donor spleen-derived CD4+ and CD8+ T cells T cells on day 10 post-transplant compared with syngenic control recipients (Fig 4B). Similarly, the numbers of donor BM-derived CD4+ and CD8+ T cells were significantly higher in BM of miHA recipients versus syngenic controls but lower in spleen on day 10 and 26 post-transplant (Fig 4B). PD-1 expressing donor spleen-derived CD4+ T cells were significantly higher in spleen only whereas PD-1 expressing donor spleen-derived CD8+ T cells were also significantly higher in the spleen and BM of miHA recipients on day 10 post-transplant compared with the syngenic control recipients (Fig 4C). PD-1 expressing CD4+ and CD8+ T cells, generated *de novo* from donor BM, were also significantly higher in the BM of miHA transplant recipient on day 10 compared with the syngenic control recipients (Fig 4C). We could not detect differences in the expression of CTLA-4 by donor spleen- and BM-derived CD4+ and CD8+ T cells in spleen or BM comparing miHA with syngenic recipients on day 10 and 26 after transplant. These data suggest that allo-reactive donor CD8+ T cells are associated with inflammation in the hematolymphoid tissues of miHA recipients, but subsequent up-regulation of PD-1 expression limits GvHD pathogenicity compared with what is seen in MHC mis-matched transplant recipients.

### Donor CD4+ and CD8+ T cells in spleen and BM re-expressed PD-1 and CTLA-4 after contraction phase

The numbers of leukocytes in the spleen rapidly expanded in allo-HSCT (~25-fold) and syn-HSCT (~200 fold) recipients on day 10 after transplant followed by a rapid decrease by day 25 post-transplant (S2 Fig). While the numbers of leukocytes in the BM expanded ~50-fold in both allo- and syn-HSCT recipients on day 10 after transplantation, the cellularity of BM did not undergo a subsequent contraction phase in either allo- or syn-HSCT recipients (S2 Fig). Since CTLA-4 causes activation-induced cell death on T cells and PD-1 functions as a cell death inducer [31, 32] we reasoned that expression of one or both molecules could play an important role in the contraction phase of donor cells in the spleen on day 25 after transplant. We next measured the kinetics of both PD-1 and CTLA-4 expressing CD4+ and CD8+ T cells in the spleen and BM of allo- and syn-HSCT recipients. The percentage of PD-1 and CTLA-4 expressing CD4+ and CD8+ donor T cells decreased considerably on day 25 but rebounded by day 103 after transplant in the spleen of both allo-HSCT and syn-HSCT recipients (Fig 2E). In contrast, the percentage of CD8+ T cells (not CD4+ T cells) expressing PD-1 and CTLA-4 decreased considerably on day 25 after transplant in the BM of both allo- and syn-HSCT recipients (Fig 2E). These data suggest that the activation status and levels of PD-1 and CTLA-4 expression on donor CD4+ and CD8+ T cells in the spleen and BM of allo-HSCT recipients GvHD are differentially regulated and overall data are summarized in Table 1.

**Table 1:** Summary of differential PD-1+ and CTLA-4+ donor spleen-derived T cells and Tregs distribution in the spleen and BM of allo-HSCT recipients.

Models	Allo-HSCT	Spleen							BM (Tibia & Femer of one hind leg)					
		Days post-BMT							Days post BMT					
		10			25		103		10		25		103	
		CD4	CD8	Treg	CD4	CD8	CD4	CD8	CD4	CD8	CD4	CD8	CD4	CD8
B6-->CB6F1	Major MHC-Mismatched	PD-1 expression												
		↑	↑	↓↓	**−**	**−**	↓↓	↓↓	↑	↑	↑	↑	↑↑	**−**
C3H.SW-->B6	Minor MHC-Mismatched	↓↓↓	cc	ND	**−**	↓↓↓	ND	ND	↓↓↓	↓↓↓	**−**	**−**	ND	ND
B6-->CB6F1	Major MHC-Mismatched	CTLA-4 expression												
		↑	↑↑	ND	↓↓	**−**	↓↓	↓↓	↑	**−**	↑	**−**	↑	**−**
C3H.SW-->B6	Minor MHC-Mismatched	**−**	**−**	ND	**−**	**−**	ND	ND	**−**	**−**	**−**	**−**	ND	ND

Comparison of absolute numbers of PD-1 and CTLA-4 expressing donor spleen-derived CD4+ and CD8+ T cells, and Tregs harvested on day 10, 25 or 26 and 103 from the spleen and BM of the major and minor MHC mis-matched allo-HSCT and syn-HSCT recipients. The symbols ↑, ↑↑, and ↑↑↑ represent the relative significance of the increase, and ↓, ↓↓, and ↓↓↓ represent the relative significance of the decrease (corresponding to p<0.05, p<0.005 and p<0.001 values (Student’s t-Test), respectively) in the mean numbers of PD-1+ and Tregs and CTLA-4+ donor spleen-derived CD4+ and CD8+ T cells in the spleen and BM of allo-HSCT recipients compared to data obtained from the spleen and BM of B6→ B6 syngeneic transplants. The “—”is for data for which there was no significant difference noted between the allo-HSCT and syn-HSCT recipients. The “ND” is for the experiments not done.

### GvHD causes reduced expression of foxp3 transcription factor by donor T cells

**Fig 5:**
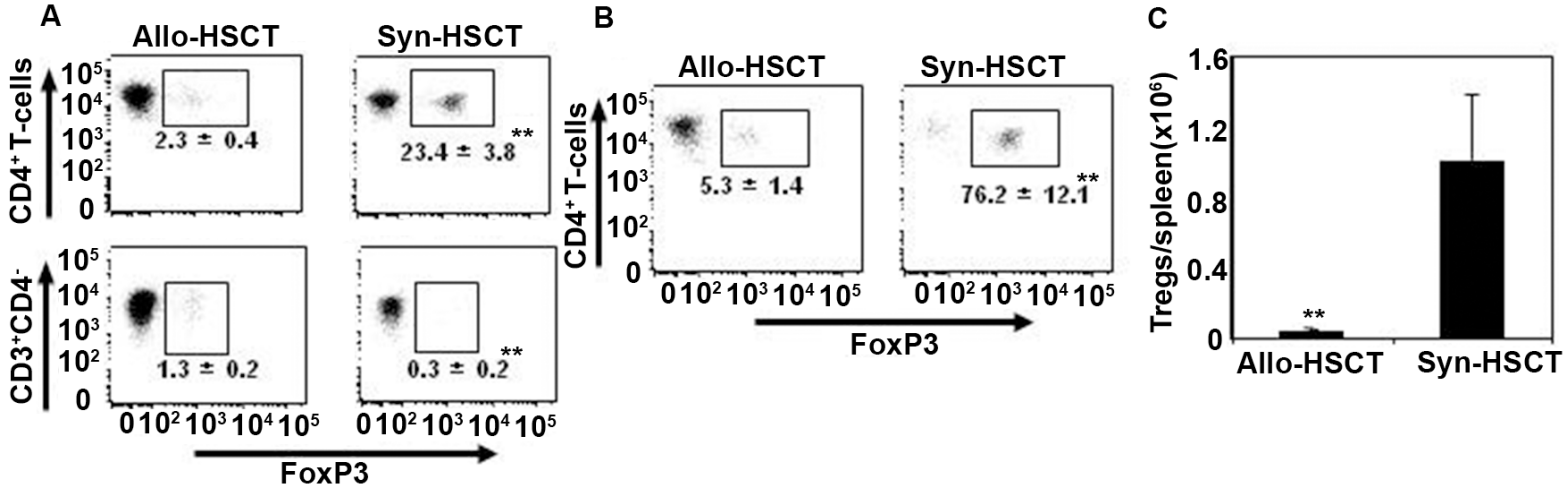
GvHD causes reduced expression of foxp3 transcription factor in donor T cells. Splenocytes were harvested from the allo-HSCT and syn-HSCT recipients (as described in Fig 1) on day 10 after transplant and expression of foxp3 in donor T cells was determined by intracellular FACS analysis. A. Foxp3 expression in CD4+ and CD4- T cell populations. B. Foxp3 expression in CD25+CD4+ regulatory T cells. C. The total numbers of Tregs per spleen. The symbols “*” and “**” represent *p* values <0.05 and <0.005, respectively, Students *t*-Test. The data are the representative of two independent experiments. 5 mice were used per group.

The transcription factor foxp3 is expressed by subsets of CD4+ and CD8+ T cells and induces negative regulatory effects on donor allo-reactive T cells. Increased foxp3 expression on donor T cells is associated with reduced levels of GvHD in both mice and in human [21, 33–38]. To explore the role of foxp3 transcription factor expressing donor T cells in the induction of GvHD, we measured foxp3 expression in CD4+ T cells and Tregs in the spleen on day 10 after transplantation in allo-HSCT recipients. The proportion of CD4+ and CD4- T cells expressing foxp3 was significantly lower (p<0.005) on day 10 post-transplant in the spleen of allo-HSCT recipients compared with syn-HSCT recipients (Fig 5A). Accordingly, the percentages of T cells that were foxp3+ and the absolute numbers of Tregs per spleen were significantly lower in allo-HSCT recipients compared with syn-HSCT recipients (Fig 5B and 5C). These data show that lower levels of foxp3 expression by donor T cells was associated with the peak expansion of activated donor T cells on day 10 post allo-HSCT.

### Inducible PD-L1 expression on host and donor tissues was insufficient to prevent GvHD in allo-HSCT recipients

**Fig 6:**
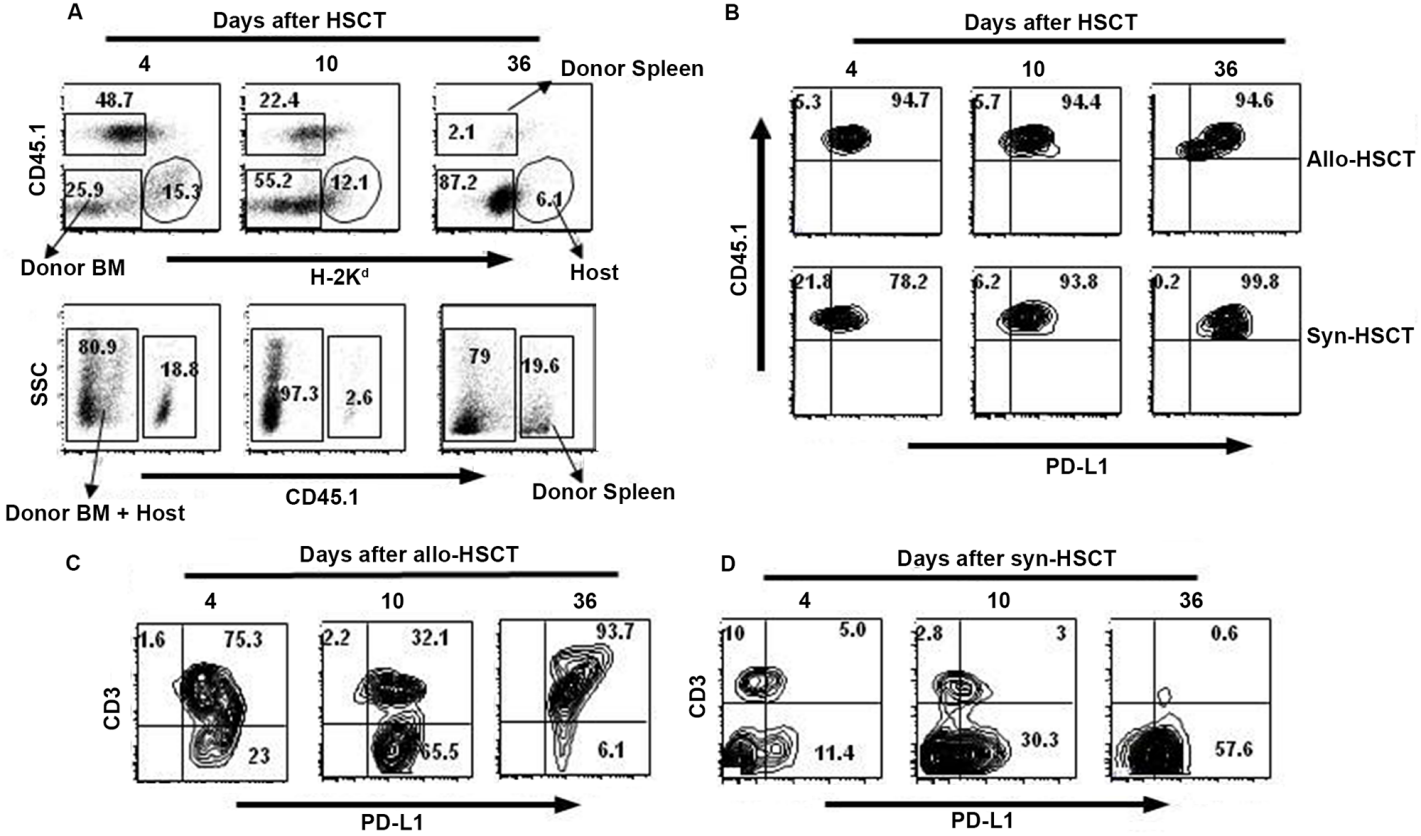
Kinetics of PD-L1 expressing donor and host cells in spleen of allo-HSCT recipients. Splenocytes were harvested on days 4, 10 and 36 after transplant from B6→ CB6F1 (allo-HSCT) and B6→ B6 (syn-HSCT) recipients and PD-L1 expression of donor spleen- (CD45.1+H-2^d^-), donor BM- (CD45.1-H-2^d^-), and host-(CD45.1-H-2^d^+), derived cells were determined. A. Representative FACS dot plots show donor chimerism in allo-HSCT (upper panel) and syn-HSCT (lower panel) recipients. B. Kinetics of PD-L1 expression by donor spleen-derived cells harvested from the spleen of allo-HSCT and syn-HSCT recipients. C. Kinetics of PD-L1 expression by host-derived CD3+ T and non-T cells harvested from the spleen of allo-HSCT recipients. D. Kinetics of PD-L1 expression by donor BM-derived CD3+ T and non-T cells in allo-HSCT recipients. The data are the representative of one independent experiment. 5 mice were used per group.

PD-L1 is expressed by various tissues of hematopoietic and non-hematopoietic origins [15] and Saha et al showed that expression of PD-L1 by host tissues is required to limit GvHD in allo-HSCT recipients [17]. To explore PD-L1 expression on donor and host cells in allo-HSCT recipients, we sacrificed allo-HSCT recipients on days 4, 10 and 36 after transplant and measured PD-L1 expression on donor spleen-, donor BM- and host-derived cells in the spleen of allo-HSCT recipients. The data were compared with similar data obtained from the syn-HSCT recipients. The percentages of donor spleen-derived (CD45.1+H-2^d^-) and host-derived (CD45.1-H-2^d^+) cells decreased over time after transplant in allo-HSCT recipients (Fig 6A, upper panel). More CD45.1+ donor cells expressed PD-L1 at all three time points in allo-HSCT recipients than syn-HSCT recipients (Fig 6B, upper panel). While most of the host-derived CD3+ T cells and CD3- non-T cells also expressed PD-L1 on day 4 post transplant, host non-T cells expressing PD-L1 eventually disappeared over time in allo-HSCT recipients (Fig 6C), with the remaining PD-L1 positive cells being donor BM-derived cells (Fig 6D). These data suggest that PD-L1 expression on host and donor hematopoietic cells was insufficient to prevent GvHD in target organs of allo-HSCT recipients.

#### Lack of PD-1:PD-L1 interaction increased activation of donor T cells but decreased foxp3 expressing Tregs

**Fig 7:**
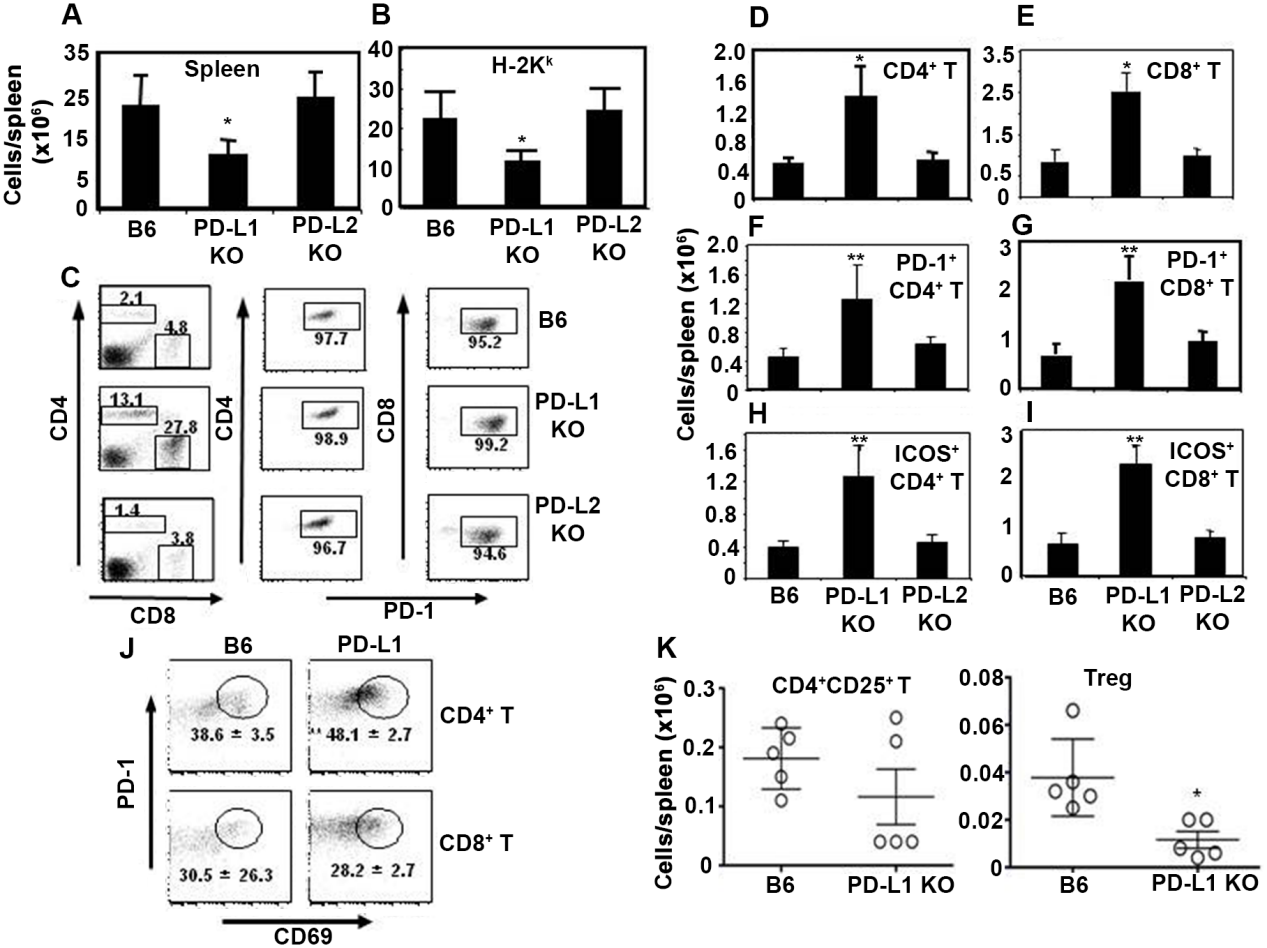
Lack of PD-L1 expression by host tissues increases numbers of activated donor T cells but decreases donor Tregs in spleen. WT B6, PD-L1 KO and DL-L2 KO mice were transplanted with 2 x10^6^ naïve congenic B10.BR T-cell enriched splenocytes (H-2^K^) and 2 x10^6^ stem cell enriched BA.B10 TCD BM (H-2^K^) as described in Materials and Methods. Recipient mice were sacrificed on day 8 after transplant. Splenocytes were harvested and immune phenotypes of donor T cells were determined by flow cytometry. A. Total numbers of leukocytes per spleen. B. Total numbers of donor-derived nucleated cells (H-2^K^+ gated) per spleen. C. H-2^K^+ cells gated FACS dot plots of (one representative mouse from each group) showing CD4+ and CD8+ T cells, and PD-1 expressing CD4+ and CD8+ T cells. D and E. Absolute numbers of H-2^K^+ gated donor CD4+ and CD8+ T cells per spleen, respectively. F and G. Absolute numbers of H-2^K^+ gated donor PD-1+CD4+ and PD-1+CD8+ T cells per spleen, respectively. H and I. Absolute numbers of H-2^K^+ gated donor ICOS-1+CD4+ and ICOS-1+CD8+ T cells per spleen, respectively. J. FACS dot plots of H-2^K^+ gated CD4+ and CD8+ T cells expressing PD-1 and CD69 on day 8 after transplant in the spleen of WT B6 and PD-L1KO allo-HSCT recipients. K. Absolute numbers of CD25+CD4+ T cells and Tregs per spleen. 5 mice were used per group. The symbol “*” indicates p value <0.05, Student’s t-Test. The data are the representative of two similar experiments.

To investigate the mechanism by which PD-L1 expression in host tissue reduces GvHD severity [17], we determined the numbers and phenotype of CD45.1+ (donor spleen-derived) CD4+ and CD8+ T cells in the spleens of B6 allo-HSCT recipients that were PD-L1 KO, PD-L2 KO or WT. As PD-L1 KO allo-HSCT recipients had severe acute GvHD and died within 10 days after transplant (S3 Fig), we sacrificed WT B6, PD-L1 KO and PD-L2 KO allo-HSCT recipients on day 8 after transplant for phenotypic analysis of splenocytes. Total numbers of nucleated cells and CD45.1+ donor cells per spleen were significantly lower in PD-L1 KO allo-HSCT recipients compared with PD-L2 KO and WT B6 allo-HSCT recipients (Fig 7A and 7B), while donor CD4+ and CD8+ T cells increased ~6-fold in the spleen of PD-L1 KO allo-HSCT recipients compared with PD-L2 KO and WT B6 allo-HSCT recipients, with >95% of all T cells expressing PD-1 in allo-HSCT recipients (Fig 7C). Accordingly, total numbers of donor CD4+ (Fig 7D) and CD8+ (Fig 7E) T cells per spleen increased significantly in PD-L1 KO allo-HSCT recipients compared with PD-L2 KO and WT B6 allo-HSCT recipients. In addition to increased PD-1 expression in donor CD4+ and CD8+ T cells in the spleen (Fig 7F and 7G), ICOS-1 expression on donor CD4+ and CD8+ T cells was also significantly increased in PD-L1 KO allo-HSCT recipients (Fig 7H and 7I). Moreover, significantly higher percentages of PD-1+ CD69+ double-positive CD4+ T cells with an activated phenotype (but not double-positive CD8+ T cells) were detected in the spleen of PD-L1 KO allo-HSCT recipients compared with WT B6 allo-HSCT recipients (Fig 7J). While the numbers of CD25+CD4+ activated T cells were similar in the spleens of PD-L1 KO allo-HSCT and WT allo-HSCT recipients (Fig 7K, left panel), significantly lower numbers of foxp3+ Tregs were seen in the spleen of PD-L1 KO allo-HSCT recipients compared with WT B6 allo-HSCT recipients (Fig 7K, right panel). Collectively, these data suggest that increased GvHD in PD-L1 KO allo-HSCT recipients is associated with increased proliferation and activation of donor CD4+ and CD8+ T cells, but not non-T cells and donor Tregs, and that up-regulation of PD-1 expression on donor T cells is independent of the expression of PD-L1 in allo-HSCT recipients.

### Severe acute GvHD in PD-L1 KO allo-HSCT recipients is associated with increased production of inflammatory cytokines

**Fig 8:**
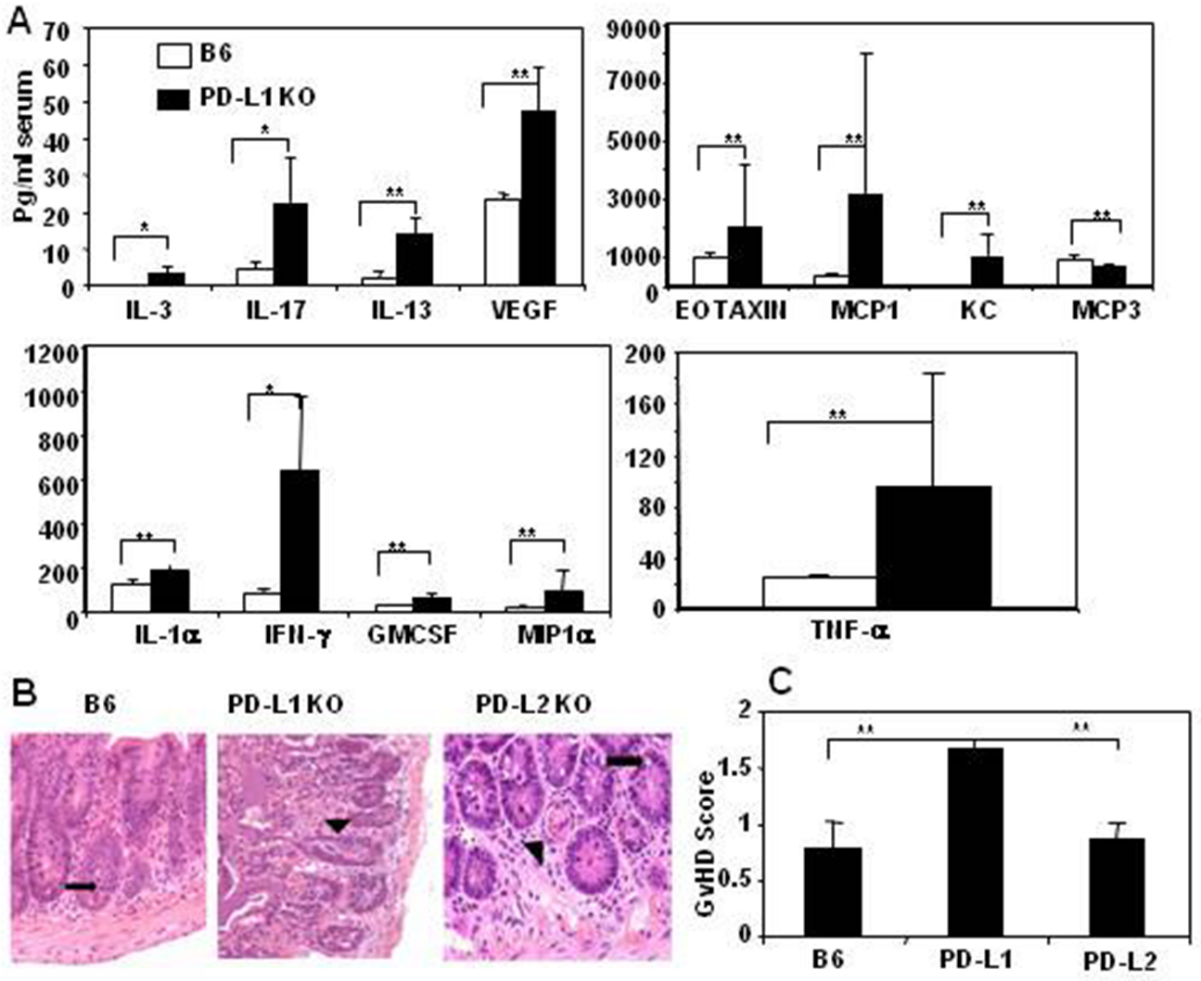
Severe acute GvHD in PD-L1 KO allo-HSCT recipients is associated with increased production of inflammatory cytokines. Liver and intestines (small and large) tissue sections were harvested from the same mice as described in Fig 7. A. Sera were collected on day 8 after transplantation and 26 Plex Luminex assay was performed to determine cytokines and chemokines serum levels as described in Materials and Methods. Cytokines and chemokines that had significantly different levels between the groups are shown. B. Represents the small intestinal histological tissue section of one representative mouse from each experimental group (n = 5). Black arrows indicate the presence of single apoptotic epithelial cells. Microscopic images of small intestine from one representative mouse of each group were taken at 200x using a Nikon Eclipse E400 microscope. C. Represents mean acute GvHD score with standard deviation that was calculated by measuring the weight loss (0–2), posture (0–2), activity (0–2), skin integrity (0–2) and histological acute GvHD scores of liver (0–1), small intestine (0–4) and large intestine (0–4). The symbols “*” and “**” represent *p* values <0.05 and <0.005, respectively, Students *t*-Test. The data are the representative of one experiment using 5 mice per group.

To determine the effect of the absence of PD-L1 expression on host cells on the immunological milieu in allo-HSCT recipients we transplanted B10.BR cells into PD-L1 KO mice and control WT B6 mice, and measured serum cytokine and chemokine levels on day 8 after transplant (S3 Fig). PD-L1 KO allo-HSCT recipients had significantly higher levels of 13 inflammatory cytokines/chemokines compared with WT B6 recipients (Fig 8A). Next, we determined the acute GvHD pathological score from formalin fixed histological tissue sections of liver, small and large intestines harvested on day 8 post-transplant from WT B6, PD-L1 KO and PD-L2 KO allo-HSCT recipients (as described in Materials and methods). PD-L1 KO allo-HSCT recipients had higher GvHD pathology as determined through their clinical GvHD scores (weight loss, posture, activity, skin integrity) and histological GvHD scores compared with WT or PD-L2 KO recipients (Fig 8B and 8C). The markedly increased acute GvHD in the absence of PD-L1 expression on host tissues compared with WT recipients indicate that PD-L1 expression by host tissue is required to limit allo-activation of donor T cells, presumably through PD1-signaling.

## Discussion

In this study, we have explored the kinetics of expression of three inhibitory immune checkpoint pathways, PD-1/PD-L1, CTLA-4, and foxp3+ Tregs, that are relevant to the control of GvHD in allo-HSCT recipients. Our data indicate that the expression levels of immune checkpoint blockade molecules and Tregs varies dynamically over time and across different hematolymphoid tissues and organs in transplant recipients. Among transplant recipients with established GvHD, the expression levels and numbers of PD-1 and CTLA-4 positive donor T cells were higher in the BM than the spleen, consistent with a recent publication showing that the BM microenvironment is relatively immune-suppressive [39]. Host APCs surviving in the spleen and BM after lethal irradiation have been shown to play a pivotal role in activating allo-reactive donor T cells that cause GvHD [40–42]. The larger numbers of mature APC in the spleen versus BM may also explain the increased susceptibility of the spleen to the effects of GvHD. The presence of GvHD in the spleen leads to marked atrophy and loss of splenic white pulp. Our data also support the data published by Zhang et al, showing that the recruitment and activation of donor allo-reactive CD8+ T cells in the liver and spleen by host DCs and macrophages was differentially regulated compared with that in lymph nodes [40].

The GvHD pathology seen in miHA mismatched allo-HSCT model has resemblance to the GvHD pathology seen in human allo-HSCT recipients [43], and the data presented in this study could provide important clues for designing therapeutic interventions to treat or prevent clinical GvHD. Significantly increased numbers of donor C3H.SW CD8+ T cells were seen in recipient BM on day 10 and in spleen on day 26 in the C3H.SW → B6 miHA mismatched allo-HSCT recipients presumably because donor CD8+ T cells mediate GvHD pathogenicity in this model (Fig 4B) [30]. Of note, in miHA mismatched model, we were unable to quantify significant numbers of CTLA-4 expressing CD4+ and CD8+ T cells in the spleen and BM through flow cytometry analysis on both day 10 and 26 after transplant, suggesting that increased PD-1 expression on donor CD8+ T cells in spleen and BM in the miHA mismatched allo-HSCT model may be sufficient to reduce GvHD pathogenicity in miHA versus MHC mis-matched allo-HSCT recipients may (Fig 4C).

PD-1 expression on T cells significantly increases in the presence of viral infection and after transplantation [17, 24, 44]. Our data confirm that PD-1 expression on CD4+ and CD8+ T cells persists in allo-HSCT recipients until 100+ days post transplant in spite of active GvHD. In the absence of PD-L1-expressing host cells the numbers of donor Tregs are decreased, inflammatory cytokine increase leading to expansion of PD-1+ donor effector T-cells and acute GvHD damage (Figs 5 and 6). Our findings in allo-HSCT recipients are consistent with those in acute LCMV-infected mice, in which PD-1+ T cells are cytolytically active [44]. Our data from allo-HSCT recipients lacking PD-L1 with severe acute GvHD and mortality are also consistent with the observation that chronically (but not acutely) LCMV-infected PD-L1 KO mice develop severe immunopathologic tissue damage and die within a few days of infection [44]. However, the effect of PD-1/PD-L1 mediated inhibitory signaling is more noticeable in tumor models as it helps tumors to escape immune surveillance by inducing apoptosis of tumor specific T cells [45]. Inhibition of either PD-1 or PD-L1 with mAbs restores the effector function of viral specific and tumor specific T cell responses, and clinical trials are currently testing PD-1 or PD-L1 blockade in cancer patients [6, 12]. Although PD-1/PD-L1 blockade can increase anti-tumor activity of donor T cells [44, 45], antibodies to PD-1/PD-L1 increase GvHD lethality and mortality in allo-HSCT recipient mice [16]. Administration of engineered soluble PDL1-Ig protein, which reduces the proliferation of T cells during transplantation, may be an alternative approach to reduce GvHD [46] although autoimmune complications will be an issue [47].

Our data showing decreased numbers of donor Tregs associated with significantly reduced expression of the intracellular foxp3 transcription factor in CD4+ T cells in the spleen of allo-HSCT recipients (Fig 5) is consistent with previously published clinical data demonstrating that allo-HSCT patients with GvHD had decreased Tregs in blood compared with patients treated with either allo-HSCT recipients without GvHD or auto-HSCT recipients [48]. Although the mechanism behind the reduced generation of Tregs in allo-HSCT recipients with GvHD is not well documented, it is conceivable that the increased production of inflammatory cytokines in allo-HSCT recipients with GvHD may prevent the generation of Tregs.

In conclusion, our data provide important information showing that expression of inducible PD-1 and CTLA-4 on donor T cells, and PD-L1 expression on both donor and host cells in allo-HSCT recipients are insufficient to prevent donor T cell mediated allo-reactivity and local GVHD-mediated inflammation in hematolymphoid organs. Although allo-HSCT recipients lacking PD-L1 had increased numbers of activated donor CD4+ and CD8+ T cells and increased GvHD severity, induction of donor Tregs did not occur. Our data suggest that activation of donor Treg through PD1 signaling by PD-L1-expressing host cells necessary to prevent severe acute GvHD. Novel approaches to regulate GvHD should focus on the preservation of PD-L1 expression on host hematopoietic and non-hematopoietic host cells of GvHD target organs alone or in combination with adoptive therapy using *ex vivo* expanded Tregs in the graft, with the goal of providing potential therapeutic benefit against GvHD.

## Supporting information

S1 Fig B6→ CB6F1 allo-HSCT recipients had signs of clinical GvHD. 5 x 10^6^ TCD BM cells plus 7.5 x 10^6^ donor splenocytes from congenic naïve B6 donors transplanted via tail vein injection into 11 Gy irradiated CB6F1 or B6 recipient mice. A and B represent the percent survival and percent weight change determined until 103 days of transplant, respectively. The data are the representative of two similar experiments using 10 mice per group.
(TIF)

S2 Fig Kinetics of donor T cells in GvHD target organs. B6→ CB6F1 allo-HSCT and B6→ B6 syn-HSCT recipients were sacrificed on day 4, 10, 25 and 103 days after transplant. Cells from spleen, BM, blood, and thymus were harvested. A, B, C, and D represent the kinetics of total nucleated cells harvested from spleen, BM, per ml blood and thymus, respectively. E. Nucleated cells harvested from spleen, BM, per ml blood and thymus on day 4 after transplant. F and G represent the kinetics of total donor spleen-derived (CD45.1^+^ gated) cells harvested from spleen and BM, respectively. The symbols “*” and “**” represent *p* values <0.05 and <0.005, respectively, Students t-Test. The data are the representative of two independent experiments. 5 mice were used per time point.
(TIF)

S3 Fig PD-L1 KO allo-HSCT recipients had more GvHD and mortality than PD-L2 KO or WT B6 allo-HSCT recipients. T-cell were enriched by depleting CD11b+CD11c+CD119+ cells from naïve congenic B10.BR (BA.B10BR) splenocytes and hematopoietic stem cells were enriched by depleting CD3+CD11b+CD11c+CD19+ cells from naïve BA.B10BR using MACS separation column. 2 x 10^6^ HSC enriched BM cells plus 2 x 10^6^ T-cells enriched splenocytes were transplanted through the tail vein of WT B6. PD-L1 KO and PD-L2 KO recipient mice one day after 11 Gy irradiation. A and B represent the percentage survival of allo-HSCT recipients until 34 days post transplant, The symbol “*” indicates *p* values <0.05, Log Rank test of groups WT B6 and PD-L2 KO HSCT recipients vs PD-L1 KO HSCT recipients. The data are the representative of two similar experiments using 5 mice per group.
(TIF)

## Abbreviations

Allo-HSCT: allogeneic hematopoietic stem cell transplantation
CTLA-4: cytotoxic T lymphocyte antigen-4
GvHD: graft-vs-host disease
PD1: program death 1
PDL-1: program death ligand 1
PDL-2: program death ligand 2
