# Supplementary figures and images for: PD-1 and CTLA-4 up regulation on donor T cells is insufficient to prevent GvHD in allo-HSCT recipients
Source: PLoS One. 2017 Sep 27;12(9):e0184254. doi: 10.1371/journal.pone.0184254 (PMC5617147; doi:10.1371/journal.pone.0184254)

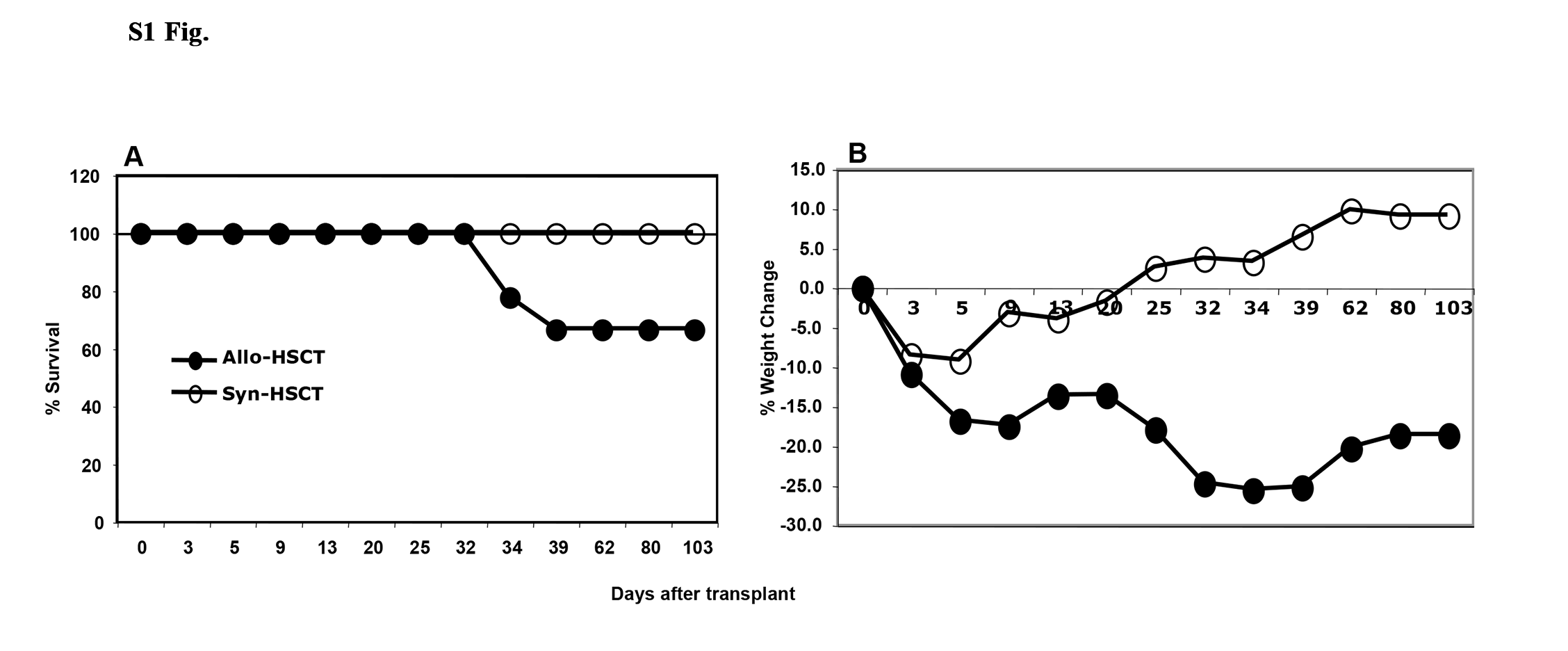

Supplement: S1 Fig — 5 x 10^6^ TCD BM cells plus 7.5 x 10^6^ donor splenocytes from congenic naïve B6 donors transplanted via tail vein injection into 11 Gy irradiated CB6F1 or B6 recipient mice. A and B represent the percent survival and percent weight change determined until 103 days of transplant, respectively. The data are the representative of two similar experiments using 10 mice per group. (TIF) [file pone.0184254.s001.tif]

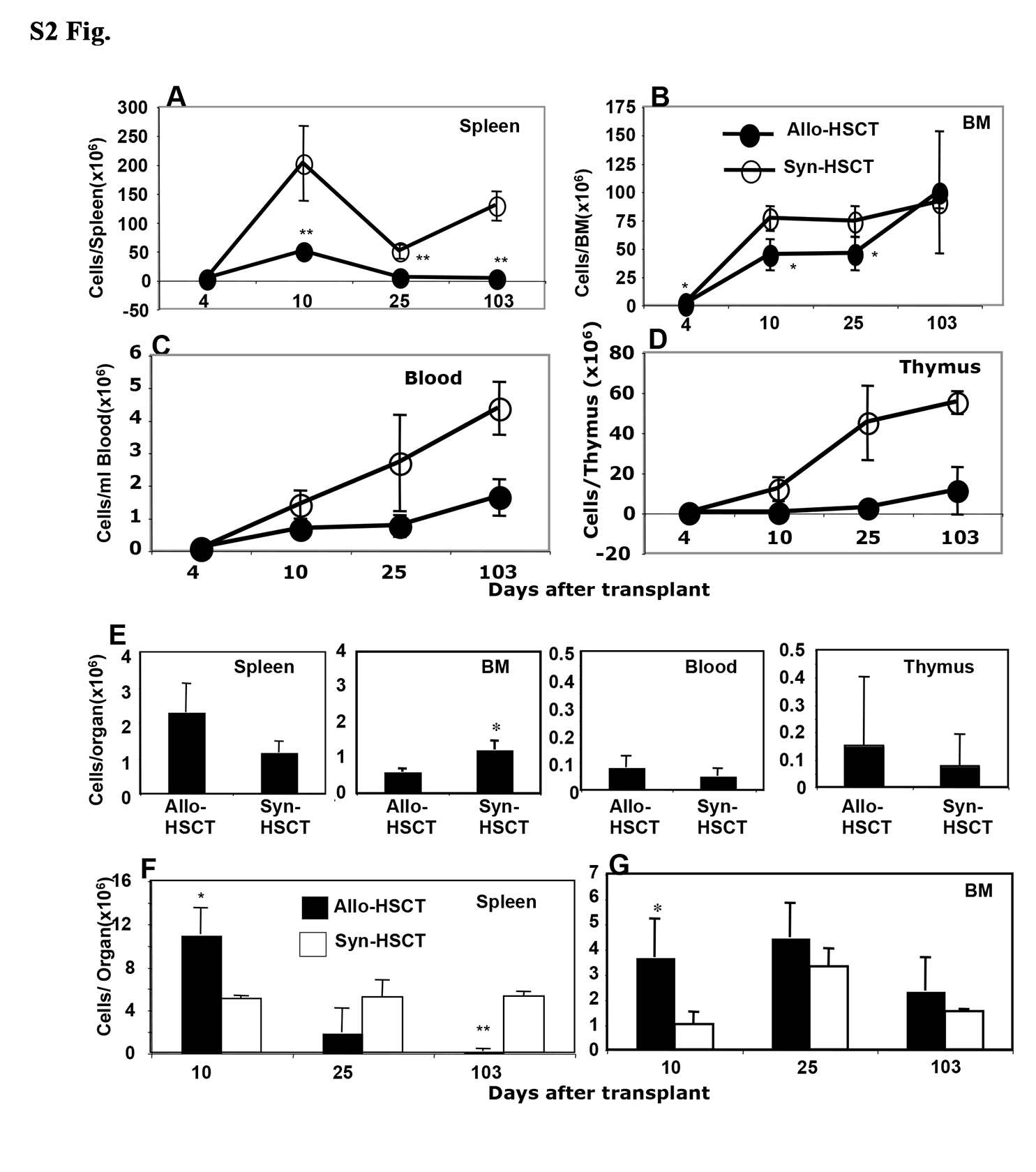

Supplement: S2 Fig — B6→ CB6F1 allo-HSCT and B6→ B6 syn-HSCT recipients were sacrificed on day 4, 10, 25 and 103 days after transplant. Cells from spleen, BM, blood, and thymus were harvested. A, B, C, and D represent the kinetics of total nucleated cells harvested from spleen, BM, per ml blood and thymus, respectively. E. Nucleated cells harvested from spleen, BM, per ml blood and thymus on day 4 after transplant. F and G represent the kinetics of total donor spleen-derived (CD45.1+ gated) cells harvested from spleen and BM, respectively. The symbols “*” and “**” represent p values <0.05 and <0.005, respectively, Students t-Test. The data are the representative of two independent experiments. 5 mice were used per time point. (TIF) [file pone.0184254.s002.tif]

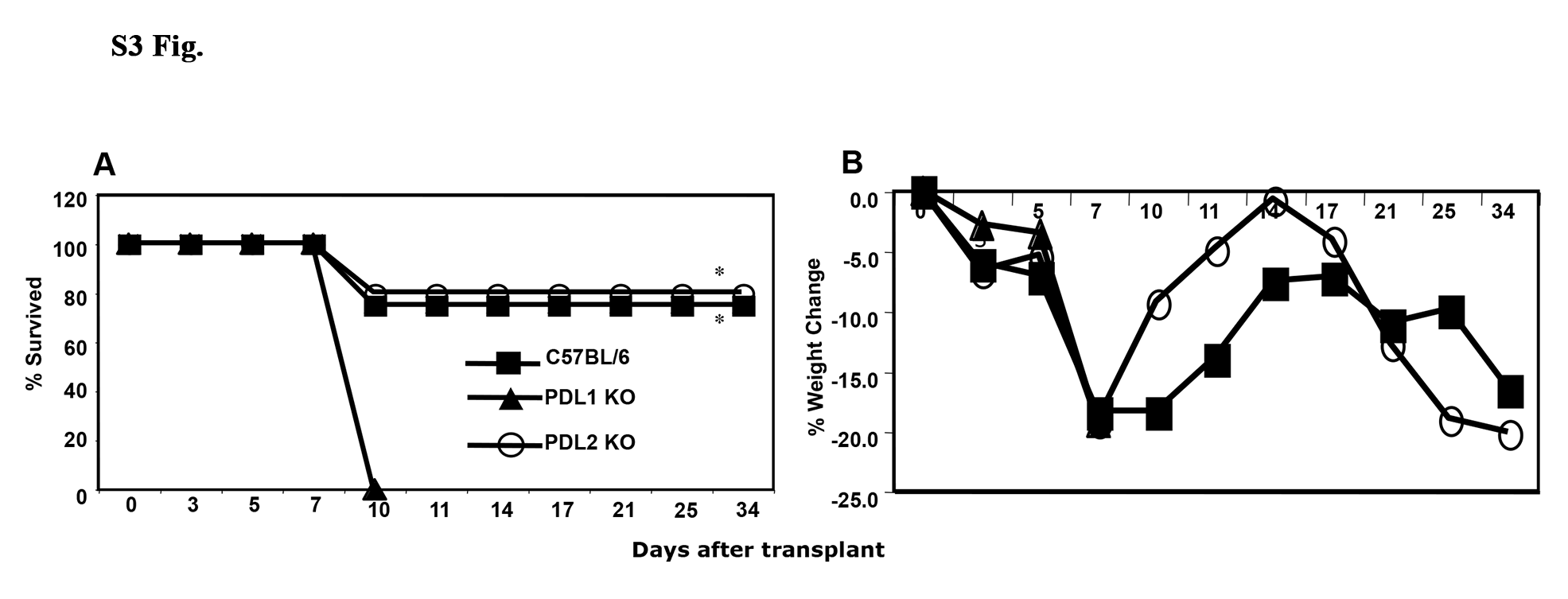

Supplement: S3 Fig — T-cell were enriched by depleting CD11b+CD11c+CD119+ cells from naïve congenic B10.BR (BA.B10BR) splenocytes and hematopoietic stem cells were enriched by depleting CD3+CD11b+CD11c+CD19+ cells from naïve BA.B10BR using MACS separation column. 2 x 10^6^ HSC enriched BM cells plus 2 x 10^6^ T-cells enriched splenocytes were transplanted through the tail vein of WT B6. PD-L1 KO and PD-L2 KO recipient mice one day after 11 Gy irradiation. A and B represent the percentage survival of allo-HSCT recipients until 34 days post transplant, The symbol “*” indicates p values <0.05, Log Rank test of groups WT B6 and PD-L2 KO HSCT recipients vs PD-L1 KO HSCT recipients. The data are the representative of two similar experiments using 5 mice per group. (TIF) [file pone.0184254.s003.tif]
